# Elevated B12/CRP Index as a Simple Prognostic Indicator in Patients with Metastatic Renal Cell Carcinoma Treated with First-Line Targeted Therapy

**DOI:** 10.3390/biomedicines14051131

**Published:** 2026-05-16

**Authors:** Oktay Halit Aktepe, Tugce Ulasli, Osman Butun, Suayib Yalcin

**Affiliations:** 1 Department of Medical Oncology, Dokuz Eylul University, Izmir 35330, Turkey; tugce.ulaslialtun@deu.edu.tr; 2 Department of Medical Oncology, Acibadem Izmir Kent Hospital, Izmir 35620, Turkey; 3 Department of Medical Oncology, Hacettepe University Cancer Institute, Ankara 06230, Turkey

**Keywords:** B12/CRP index, C-reactive protein, metastatic renal cell carcinoma, prognosis, vitamin B12

## Abstract

**Background/Objectives:** The vitamin B12 (VB12)/C-reactive protein (CRP) index (BCI), a clinically derived index calculated as serum VB12 multiplied by CRP, has shown prognostic value in several cancers. However, its association with survival outcomes in metastatic renal cell carcinoma (mRCC) remains unclear. Therefore, the aim of the present study was to evaluate the prognostic significance of BCI in patients with mRCC treated with targeted therapy. **Methods:** The BCI was calculated as serum VB12 concentration (pg/mL) × serum CRP concentration (mg/L). The patients were categorized into two BCI prognostic subgroups, high BCI (BCI > 40,000) and low BCI (≤40,000). Survival differences between prognostic subgroups were measured using the Kaplan–Meier method with a log-rank test. Univariate and multivariable analyses were used to determine the association between the selected variables and survival outcomes. **Results:** We included 213 patients with mRCC, with a median follow-up time of 76 months. The median progression-free survival (PFS) and overall survival (OS) were 10.9 months and 47.7 months, respectively. Patients with high BCI had poorer PFS and OS times than those with low BCI (7.8 months vs. 12.6 months, *p* = 0.002 for PFS; 22.6 months vs. 68 months, *p* < 0.001 for OS, respectively). After adjusting for potential confounders, high BCI remained independently associated with poorer PFS and OS (hazard ratio [HR]: 2.40, 95% confidence interval [CI] 1.35–4.26, *p* = 0.003 for PFS; HR 2.01, 95% CI 1.40–2.88, *p* < 0.001 for OS). **Conclusions:** BCI appears to be a promising prognostic biomarker in patients with mRCC treated with first-line targeted therapy. However, its applicability to immune checkpoint inhibitor-based or combination regimens requires prospective validation.

## 1. Introduction

Renal cell carcinoma (RCC) is a significant cause of cancer-related morbidity and mortality worldwide [1]. Despite advancements in targeted therapies and immune checkpoint inhibitors (ICIs), the five-year survival rate for metastatic RCC (mRCC) is only 12%, indicating a dismal prognosis [2]. Thus, there is an urgent need for reliable prognostic biomarkers that can guide treatment decisions and improve patient management, in addition to the currently used prognostic scoring system, the International mRCC Database Consortium (IMDC). The IMDC risk model, although widely used in clinical practice, is largely based on clinicopathologic features and routine laboratory parameters and does not directly capture tumor-driven systemic inflammation or host metabolic/nutritional status. In the contemporary mRCC landscape, where outcomes are also influenced by treatment sequencing and variability in post-progression survival, such models may not fully reflect the biological heterogeneity of the disease. Accordingly, there is increasing interest in identifying additional biomarkers that more precisely capture systemic inflammatory and metabolic alterations.

Vitamin B12 (VB12) and C-reactive protein (CRP) are two biomarkers that have gained attention in predicting survival outcomes in various cancers, including RCC [3,4,5,6,7]. VB12 plays a critical role in DNA synthesis and cellular metabolism [8], while CRP serves as an acute-phase marker that reflects systemic inflammation [9]. Emerging evidence suggests that systemic inflammation, as indicated by elevated CRP levels, and VB12 deficiency may contribute to tumor proliferation and metastasis [10,11], adversely affecting survival outcomes in cancer patients. However, the association between VB12 and CRP is intriguing, as the VB12/CRP index (BCI) could serve as a composite marker in predicting survival outcomes of several cancers [12,13,14], suggesting the significance of the nutritional status and inflammatory response within the tumor microenvironment on survival. However, limited evidence exists about the combined effect of these two biomarkers in predicting survival outcomes of mRCC. We therefore aimed to investigate the prognostic significance of BCI in predicting survival outcomes specifically in patients with metastatic RCC treated with first-line targeted therapy.

## 2. Materials and Methods

### 2.1. Patients

This multicenter retrospective study was conducted in mRCC patients who visited the Department of Medical Oncology of Dokuz Eylul University (Izmir, Turkey) and the Department of Medical Oncology of Hacettepe University Cancer Institute (Ankara, Turkey) between January 2015 and January 2025. All patients received first-line targeted therapy for metastatic disease, consisting of pazopanib, sunitinib, or cabozantinib, and no patient had a history of neoadjuvant or adjuvant systemic therapy. Baseline demographic and clinicopathologic characteristics, including age, gender, histological type, metastatic region sites, and the type of systemic therapy received, were collected from the electronic records of the centers participating in the study. We included patients aged 18 years or older with a histologically confirmed RCC diagnosis and available data on baseline serum VB12 and CRP levels within three months before the initiation of targeted therapies, and excluded those with (1) prior gastrectomy history, (2) usage of oral or injection forms of VB12 within the previous 2 years, (3) known conditions affecting VB12 absorption including pernicious anemia, (4) infectious diseases treated with antibiotics within 3 months, and (5) acute or chronic non-malignant liver disease (e.g., chronic hepatitis, cirrhosis, or active hepatic inflammation/cholestasis); the presence of liver metastases was not an exclusion criterion. Patients were stratified according to the IMDC prognostic scoring system and categorized into favorable, intermediate, and poor risk groups [15]. The BCI was calculated by multiplying serum VB12 concentration (pg/mL) with CRP concentration (mg/L). Baseline serum VB12 and CRP values were obtained from routine laboratory records at the participating centers. All measurements were performed in certified clinical laboratories using standardized methods according to institutional laboratory procedures. For each patient, the most recent pretreatment VB12 and CRP values measured within 3 months before the initiation of first-line targeted therapy were used for BCI calculation. The patients were evaluated in two prognostic subgroups according to BCI levels: high BCI (BCI > 40,000) and low BCI (≤40,000), as in the previous study by Couderc et al. [16].

### 2.2. Statistical Analysis

Categorical variables were summarized as frequencies and percentages, whereas continuous variables were reported as medians with interquartile ranges (IQRs). Progression-free survival (PFS) was calculated from the start of targeted therapy to radiological/clinical disease progression or death from any cause. Patients who had not experienced progression were censored at their last disease evaluation. Overall survival (OS) was calculated from the start of targeted therapy to death from any cause, and patients who were alive at the time of analysis were censored at the date of last follow-up. Survival outcomes were estimated using the Kaplan–Meier method, and differences between survival curves were assessed with the log-rank test. The prognostic impact of each variable was first evaluated using univariate Cox proportional hazards regression. Variables with a *p*-value of ≤0.20 in univariate analyses were subsequently included in the multivariable Cox regression model. The proportional hazards assumption for BCI was evaluated by adding a time-dependent interaction term between BCI status and the natural logarithm of OS time to the Cox regression model. To further assess the robustness of the findings, additional multivariable Cox regression analyses were performed using BCI as a continuous log-transformed variable. Receiver operating characteristic (ROC) analysis was performed to compare the discriminatory performance of the IMDC-only model and the IMDC plus BCI model for OS, and the area under the curve (AUC) was calculated for each model. Interaction terms between BCI status and first-line targeted therapy were also included in Cox regression models to evaluate whether the association between BCI and survival outcomes differed according to treatment type. OS was defined as the primary endpoint of the study, whereas PFS, subgroup analyses, interaction analyses, and sensitivity analyses were regarded as secondary or exploratory analyses. Because multiple comparisons were performed, particularly across secondary endpoints and subgroup analyses, these findings were interpreted as exploratory and hypothesis-generating rather than confirmatory. No formal multiplicity adjustment was applied to secondary and subgroup analyses. All statistical analyses were performed using SPSS version 27.0 (IBM Corp., Armonk, NY, USA), and *p*-values < 0.05 were considered statistically significant.

## 3. Results

### 3.1. Patient Characteristics

Table 1 presents the baseline patient characteristics of the whole cohort and stratified groups according to BCI levels. A total of 213 patients were included; the median age was 63 years, with males accounting for 72.8% of the cohort. The majority of patients had clear-cell RCC (77%) and grade III–IV tumors (67.1%). The most common metastatic site was lung (69.5%), followed by bone (28.2%) and liver (19.2%); brain metastases were less common (7%). The percentages of patients classified into favorable, intermediate, and poor risk according to IMDC score were 14.1%, 54.9%, and 31%, respectively. Baseline patient characteristics were generally similar between the BCI groups. There was only a significant difference in IMDC risk groups between low BCI and high BCI groups (favorable, 17.5% vs. 9.7%; intermediate, 60% vs. 48.4%; poor, 22.5% vs. 41.9%, respectively). Renal function and nephrectomy status were comparable between the BCI groups. The median estimated glomerular filtration rate (eGFR) was 65 mL/min/1.73 m^2^ in the overall cohort, with similar values in the low- and high-BCI groups (64 vs. 67 mL/min/1.73 m^2^, respectively; *p* = 0.129). Likewise, nephrectomy status did not differ significantly between the BCI groups. In the overall cohort, most patients had undergone nephrectomy (90.1%). The proportion of patients who had undergone nephrectomy was similar between the low- and high-BCI groups (90.0% vs. 90.3%, respectively; *p* = 0.938).

Considering the types of first-line treatment, pazopanib was the most common targeted therapy agent (49.3%), followed by sunitinib (41.3%), and cabozantinib (9.4%). A total of 146 patients (68.5%) received second-line therapy (nivolumab, 35.2%; axitinib, 16.9%; everolimus, 14.6%, cabozantinib, 1.9%). The percentages of patients who received third and fourth-line therapy were 30.5% (nivolumab, 7%; axitinib, 16.4%; everolimus, 3.3%, cabozantinib, 3.8%), and 14.1% (nivolumab, 2.3%; axitinib 4.2%; everolimus, 4.2%, cabozantinib, 3.3%), respectively. Treatment response to first-line targeted therapy differed significantly according to BCI status. Partial response was more frequent in the low-BCI group than in the high-BCI group (62.5% vs. 44.1%), whereas stable disease (25.0% vs. 36.6%) and progressive disease (12.5% vs. 19.4%) were more common in the high-BCI group (*p* = 0.028). As expected, baseline serum VB12 and CRP levels were higher in the high-BCI group than in the low-BCI group. The median VB12 level was 191 pg/mL in the low-BCI group and 375 pg/mL in the high-BCI group, whereas the median CRP level was 50 mg/L and 124 mg/L, respectively.

To further evaluate potential baseline imbalance within IMDC risk strata, we compared clinicopathological characteristics between low- and high-BCI groups separately in the IMDC favorable/intermediate-risk and poor-risk subgroups. In both strata, baseline variables, including age, gender, nephrectomy status, histology, tumor grade, eGFR, and metastatic sites, were generally well balanced between BCI groups, with no statistically significant differences observed (Supplementary Tables S1 and S2).

### 3.2. Survival Outcomes

**Figure 1:**
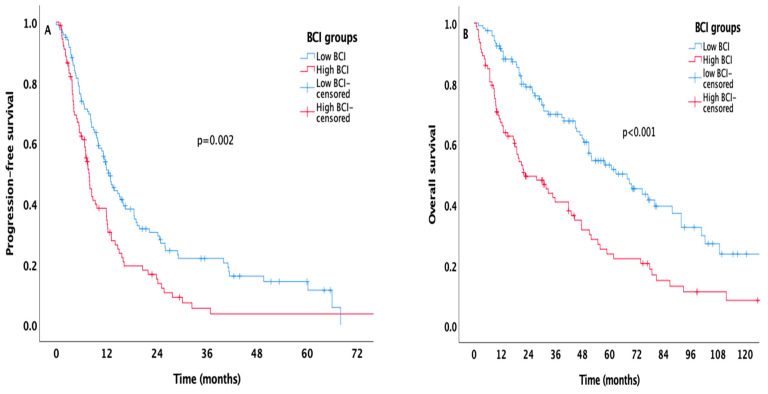
Kaplan–Meier curves estimating PFS (**A**) and OS (**B**) in all the patients stratified according to the BCI level.

A total of 132 patients (62%) died during the median follow-up time of 76 months (95% CI 65.3–86.8). The median PFS and OS of the overall population were 10.9 months and 47.7 months, respectively. Patients with elevated BCI had shorter PFS than those with low BCI (7.8 months, 95% confidence interval [CI] 6.8–8.8 vs. 12.6 months, 95% CI 10.5–14.7, *p* = 0.002, respectively, Figure 1A). The median OS of patients with low BCI was approximately threefold higher than that of the high-BCI group (68 months, 95% CI 48.5–87.4 vs. 22.6 months, 95% CI 10.6–34.7, *p* < 0.001, respectively, Figure 1B).

**Figure 2:**
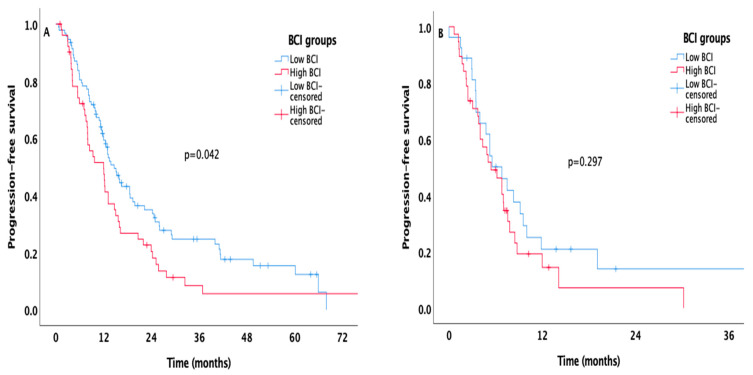
Kaplan–Meier curves estimating PFS in patients with IMDC favorable/intermediate (**A**) and poor risk (**B**) stratified according to the BCI level.

**Figure 3:**
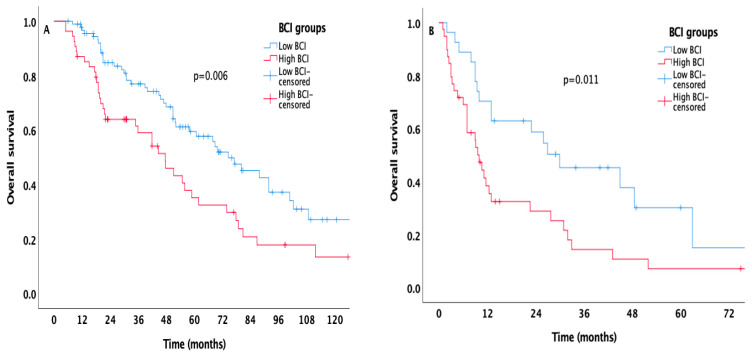
Kaplan–Meier curves estimating OS in patients with IMDC favorable/intermediate (**A**) and poor risk (**B**) stratified according to the BCI level.

Regarding PFS and OS times of IMDC risk groups stratified according to BCI levels, patients were divided into two groups, favorable/intermediate and poor risk. In patients with favorable/intermediate-risk group, high BCI was associated with worsened PFS (12 months, 95% CI 7.7–16.2 for high BCI group vs. 14.6 months, 95% CI 11.8–17.5 for low BCI group, *p* = 0.042, Figure 2A) while in patients with poor-risk, there was no significant difference between BCI prognostic subgroups (high BCI, 5.4 months, 95% CI 2.5–8.2 vs. 6.8 months 95% CI 4.1–9.4, *p* = 0.297, Figure 2B). Patients with low BCI had superior median OS times compared to those with high BCI in both favorable/intermediate and poor risk (74.7 months, 95% CI 56.3–93 vs. 47.7 months, 95% CI 33.5–61.8, *p* = 0.006 for favorable/intermediate-risk, Figure 3A; 30 months, 95% CI 9.2–50.7 vs. 10 months 95% CI 7.6–12.3, *p* = 0.011 for poor risk, Figure 3B, respectively).

As presented in Table 2 and Table 3, univariate and multivariable Cox analyses demonstrated the association of selected clinicopathological variables, including BCI, with PFS and OS. In the univariate Cox analysis for PFS, high BCI was significantly associated with poorer PFS (Hazard ratio [HR]: 1.62, 95% CI 1.19–2.21, *p* = 0.002). IMDC-intermediate risk (HR: 1.73, 95% CI 1.07–2.82, *p* = 0.025) and IMDC-poor risk (HR: 3.92, 95% CI 2.31–6.67, *p* < 0.001) were also significantly associated with poorer PFS. Nephrectomy status was significantly associated with PFS (HR: 0.23, 95% CI 0.08–0.66, *p* = 0.006), whereas lung metastasis (HR: 0.75, 95% CI 0.54–1.04, *p* = 0.091) and liver metastasis (HR: 1.40, 95% CI 0.96–2.05, *p* = 0.077) showed borderline associations with PFS. In the multivariable Cox analysis for PFS, high BCI remained independently associated with poorer PFS (HR: 2.40, 95% CI 1.35–4.26, *p* = 0.003). Nephrectomy status was also independently associated with PFS (HR: 0.12, 95% CI 0.03–0.41, *p* < 0.001). Liver metastasis showed a borderline association with PFS (HR: 1.86, 95% CI 1.00–3.45, *p* = 0.050), while lung metastasis was not statistically significant in the multivariable model (HR: 0.61, 95% CI 0.33–1.14, *p* = 0.127). IMDC risk classification remained independently associated with PFS (overall *p* < 0.001), with increased risk observed in the intermediate-risk group (HR: 2.22, 95% CI 1.08–4.57, *p* = 0.030) and poor-risk group (HR: 8.14, 95% CI 3.33–19.89, *p* < 0.001).

In the univariate Cox analysis for OS, non-clear cell histology (HR: 1.59, 95% CI 1.08–2.35, *p* = 0.018), bone metastasis (HR: 1.57, 95% CI 1.09–2.28, *p* = 0.015), brain metastasis (HR: 1.98, 95% CI 1.09–3.60, *p* = 0.024), and high BCI (HR: 2.18, 95% CI 1.54–3.07, *p* < 0.001) were significantly associated with poorer OS. IMDC-intermediate risk (HR: 2.19, 95% CI 1.15–4.17, *p* = 0.016) and IMDC-poor risk (HR: 6.69, 95% CI 3.41–13.14, *p* < 0.001) were also significantly associated with poorer OS. Treatment type was not significantly associated with OS in the univariate analysis (overall *p* = 0.163; sunitinib vs. pazopanib: HR 1.30, 95% CI 0.91–1.86, *p* = 0.146; cabozantinib vs. pazopanib: HR 1.73, 95% CI 0.87–3.40, *p* = 0.112). In the multivariable Cox analysis for OS, non-clear cell histology (HR: 1.85, 95% CI 1.20–2.85, *p* = 0.005), bone metastasis (HR: 1.55, 95% CI 1.05–2.27, *p* = 0.025), and brain metastasis (HR: 2.02, 95% CI 1.09–3.75, *p* = 0.025) remained independently associated with poorer OS. High BCI was also an independent prognostic factor for poorer OS (HR: 2.01, 95% CI 1.40–2.88, *p* < 0.001). In addition, IMDC risk classification was independently associated with OS (overall *p* < 0.001), with increased risk observed in the intermediate-risk group (HR: 2.34, 95% CI 1.21–4.52, *p* = 0.011) and poor-risk group (HR: 6.60, 95% CI 3.23–13.11, *p* < 0.001). Treatment type was included in the multivariable OS model but was not statistically significant. The discriminatory performance of IMDC alone and IMDC plus BCI for OS was also evaluated using ROC analysis. The AUC increased from 0.633 (95% CI 0.556–0.710, *p* = 0.001) for the IMDC-only model to 0.674 (95% CI 0.600–0.748, *p* < 0.001) after adding BCI.

Given the heterogeneity of first-line targeted therapy in the study cohort, we further examined whether the association between BCI and survival outcomes differed according to treatment type. For this purpose, interaction terms between BCI status and first-line targeted agent were added to the Cox regression models, with pazopanib used as the reference treatment category. In the OS model, high BCI remained significantly associated with poorer OS after adjustment for IMDC risk group, bone metastasis, brain metastasis, histology, treatment type, and interaction terms (HR: 3.04, 95% CI 1.74–5.32; *p* < 0.001). A significant interaction was observed between BCI status and sunitinib treatment compared with pazopanib (BCI × sunitinib: HR: 0.44, 95% CI 0.20–0.93; *p* = 0.033), whereas the interaction between BCI status and cabozantinib treatment was not significant (BCI × cabozantinib: HR: 0.95, 95% CI 0.23–3.88; *p* = 0.950). Additionally, treatment-stratified Cox analyses were performed according to first-line targeted therapy. In the pazopanib subgroup, high BCI was significantly associated with OS after adjustment for IMDC risk group, bone metastasis, brain metastasis, and histology (HR 2.90, 95% CI 1.58–5.31; *p* < 0.001). In the sunitinib subgroup, the association between high BCI and OS was not statistically significant (HR 1.48, 95% CI 0.88–2.51; *p* = 0.136). In the cabozantinib subgroup, the association between high BCI and OS was also not statistically significant (HR 2.12, 95% CI 0.35–12.81; *p* = 0.410).

The proportional hazards assumption for BCI was evaluated using a time-dependent BCI × log(OS time) term in the Cox model. This term was not statistically significant (*p* = 0.685). As shown in Table 4, to further assess the robustness of our findings, we performed additional multivariable Cox regression analyses using BCI as a continuous log-transformed variable. In the OS model, log-transformed BCI remained independently associated with poorer OS after adjustment for treatment type, histology, bone metastasis, brain metastasis, and IMDC risk group (HR: 1.37, 95% CI: 1.14–1.65; *p* < 0.001).

## 4. Discussion

Recent studies have suggested that the BCI may be associated with survival outcomes in patients with advanced or older cancer populations [12,16,17]. However, evidence regarding its prognostic relevance in mRCC remains limited. To our knowledge, the present study is the first to evaluate the association between BCI and survival outcomes in patients with mRCC treated with first-line targeted therapy. In this cohort, high BCI was associated with significantly shorter PFS and OS, and this association remained independent after adjustment for established clinicopathological prognostic factors. The prognostic relevance of BCI was particularly consistent for OS, which was defined as the primary endpoint of the study. In the multivariable OS model, high BCI remained independently associated with poorer survival, and this finding was further supported by the sensitivity analysis using log-transformed BCI as a continuous variable. In addition, no apparent violation of the proportional hazards assumption was observed for BCI in the OS model, supporting the stability of the Cox model estimate over time. These findings suggest that BCI may serve as a simple clinically derived prognostic index in patients with mRCC receiving first-line targeted therapy. Therefore, the present findings should be interpreted within this treatment context and should not be extrapolated to ICI-based or combination regimens.

VB12 is a cofactor in the synthesis of methionine from homocysteine, a critical process for DNA methylation. Adequate VB12 levels can prevent mutations and genomic instability by playing an essential role in the synthesis and methylation of desoxyribonucleic acid (DNA) [18], indicating a negative association between serum VB12 levels and the risk of cancer development. Carmel et al. in 1977 studied the association of VB12 with clinical parameters and survival outcomes of solid tumors and reported that elevated VB12 levels were related to the presence of distant metastasis, particularly liver, and early death [19]. It was suggested that higher VB12 levels resulted from increased production of transcobalamins by the tumor and elevated transcobalamin I (TCN1) levels due to hyperleukocytosis [19,20,21]. TCN1, a member of the VB12-binding protein family, takes part in VB12 homeostasis and is an indicator of worse survival in various cancers [22,23,24]. Zhu et al. demonstrated that TCN1 expression was related to the presence of more aggressive tumor, increased tumor markers, and regional lymph node metastasis in patients with colorectal tumors [25]. VB12 is also involved in hematopoiesis. Deficiencies in VB12 can result in immune dysfunction by reducing T cell count and natural killer cell activity and increasing cluster of differentiation (CD)4^+^/CD8^+^ ratio [26,27]. All these data indicate that VB12 levels could play a critical role in cancer development, progression, and survival.

CRP is an acute-phase protein synthesized by the liver in response to inflammation [9], and its levels frequently rise in the presence of various malignancies, including RCC [28,29]. In mRCC, elevated CRP level, reflecting an underlying inflammatory state, is not only a marker of systemic inflammation but also indicative of a more aggressive tumor biology [30]. Elevated CRP may be associated with increased production of pro-inflammatory cytokines such as TNF-α and IL-6, which are known to promote tumor cell proliferation, invasion, and angiogenesis [11]. Yano et al. investigated the prognostic importance of serum baseline CRP in predicting OS of patients with clear cell and non-clear cell mRCC receiving nivolumab plus ipilimumab and showed that elevated CRP levels were associated with inferior OS [31]. Also, it was demonstrated that elevated CRP level was an independent prognosticator in predicting survival in mRCC patients treated with targeted therapy [32,33].

In mRCC, a broad range of inflammation-based biomarkers have been investigated for prognostication. Beyond CRP alone, peripheral blood count-derived indices such as the neutrophil-to-lymphocyte ratio, platelet-to-lymphocyte ratio, and systemic immune-inflammation index have been repeatedly linked to survival outcomes, supporting the relevance of host inflammatory status in this disease [34,35,36]. This association is biologically plausible, as RCC is a highly vascular and immunogenic malignancy in which inflammatory signaling may contribute to angiogenesis, immune dysregulation, cancer-related cachexia, impaired treatment tolerance, and ultimately poorer survival [37,38,39,40]. However, although these markers provide useful information regarding inflammatory burden, they may not fully capture the broader host-related biological alterations that accompany advanced cancer, including metabolic and nutritional disturbances. In this context, BCI may provide complementary clinical prognostic information by combining two serum-based parameters, VB12 and CRP, in a single index. Importantly, elevated serum VB12 should not be interpreted simply as an indicator of adequate nutritional reserve; in patients with cancer, high circulating VB12 may instead be associated with increased levels of cobalamin-binding proteins, hepatic release or impaired hepatic clearance, inflammatory conditions, and tumor-associated alterations in transcobalamin-related pathways [21,41,42]. However, because the present study did not include molecular or mechanistic analyses, BCI should be interpreted as a clinically derived prognostic index rather than a direct mechanistic biomarker of inflammation, metabolism, or tumor biology. Therefore, any biological interpretation of BCI in mRCC remains hypothesis-generating.

The prognostic framework of mRCC has also expanded beyond conventional clinicopathological models and peripheral inflammatory markers to include immune-related and treatment-associated molecular biomarkers. Lu et al. reported that programmed cell death ligand 1 and programmed cell death ligand 2 expression had prognostic and clinicopathological relevance in RCC, highlighting the potential contribution of immune-related tumor features to risk stratification [43]. In addition, Peng et al. identified immune infiltration-related molecular signatures associated with sunitinib response in clear-cell RCC, suggesting that treatment-related molecular and immune features may influence resistance, prognosis, and disease progression [44]. Together, these findings emphasize that prognostication in mRCC is multifactorial and should integrate clinical risk models, tumor-related molecular features, treatment-associated factors, and host-related parameters. Within this framework, BCI may be regarded as a clinically derived prognostic index that provides additional host-related information, rather than a replacement for established clinical models or emerging molecular and immune-related biomarkers.

Geissbühler et al. identified the BCI as an independent factor of worse survival in cancer patients requiring palliative care and categorized patients into different prognostic groups according to the BCI levels as group I (BCI ≤ 10,000), group II (BCI 10,001–40,000), and group III (BCI > 40,000). The survival rates of group I, II, and III at 3 months were 52.8%, 27.5% and 9.4%, respectively [17]. However, the association of the BCI with survival outcomes of cancer patients has been evaluated in few studies. Kelly et al. tested the prognostic value of the BCI in palliative care cancer patients and showed that patients with an elevated BCI (>40,000) had shorter OS than those with lower BCI values [12]. Couderc et al. identified BCI as a determinant of poor prognosis in older cancer patients aged ≥ 70 years [16]. Similar to these findings, our study showed that BCI level > 40,000 was independently associated with worse PFS and OS in patients with mRCC treated with first-line targeted therapy.

Given the relatively small proportion of favorable-risk patients in our cohort and the predominance of intermediate-risk disease, we combined favorable and intermediate IMDC categories only for the Kaplan–Meier subgroup analyses to preserve statistical power and avoid unstable estimates. Importantly, this subclassification was limited to these exploratory plots. In contrast, IMDC risk was incorporated using the conventional three-category classification (favorable, intermediate, and poor) in the multivariable Cox models to ensure appropriate adjustment for baseline prognostic differences. Moreover, given the significant imbalance in IMDC risk distribution between BCI groups, we also performed an IMDC-stratified Cox regression; high BCI remained independently associated with worse OS, indicating that the prognostic effect of BCI was not solely explained by baseline IMDC risk.

The current study has several limitations when interpreting our findings regarding the prognostic value of the BCI on PFS and OS in mRCC. First, because this was a retrospective study conducted at two tertiary referral centers, the cohort may be subject to selection and referral bias (e.g., overrepresentation of patients fit for targeted therapy and with available baseline VB12/CRP measurements), and patients with missing data or specific comorbid conditions were excluded; therefore, the study population may not fully reflect the broader mRCC population, limiting the generalizability of our findings. Second, our cohort was predominantly composed of IMDC intermediate-risk patients, which may further limit extrapolation of the prognostic performance of BCI; thus, external validation in independent cohorts with more homogeneous or more balanced IMDC risk distributions is warranted. Third, while we identified the BCI as a prognostic factor of poorer survival outcomes in mRCC, the underlying biological mechanisms linking VB12 and CRP to tumor biology and survival remain unclear. Further studies are necessary to elucidate these mechanisms. Fourth, although baseline eGFR was comparable between the BCI groups, detailed data on specific comorbidities, including chronic kidney disease, ischemic heart disease, heart failure, hypertension, and diabetes mellitus, were not consistently available in the retrospective records and therefore could not be incorporated into the multivariable Cox models. Fifth, treatment sequencing, year of treatment, and later-line therapies could not be fully controlled because of the retrospective design, and may therefore have contributed to residual confounding. Additionally, we were unable to evaluate the association between the BCI and survival outcomes in patients receiving ICIs or its combination with targeted therapy due to the past reimbursement policies in our country. Thus, future prospective, multicenter studies should evaluate the prognostic and potential predictive value of BCI in patients receiving ICI-based regimens. Finally, the >40,000 BCI threshold was derived primarily from older and/or palliative care populations and has not been formally validated in younger mRCC cohorts. Therefore, its applicability in younger mRCC populations requires prospective external validation.

## 5. Conclusions

Our findings indicate that BCI may have prognostic relevance for both PFS and OS in patients with mRCC treated with first-line targeted therapy. BCI should be interpreted as a clinically derived prognostic index rather than a mechanistic biomarker, and its applicability to ICI-based or combination regimens requires prospective validation.

## Figures and Tables

**Table 1:** Baseline patients’ characteristics stratified according to BCI levels.

Characteristics	All Patients (*n* = 213)	Low BCI (*n* = 120)	High BCI (*n* = 93)	*p* Value
Age (years)				0.363
<65	140 (65.7%)	82 (68.3%)	58 (62.4%)	
≥65	73 (34.3%)	38 (31.7%)	35 (37.6%)	
Gender				0.254
Female	58 (27.2%)	29 (24.2%)	29 (31.2%)	
Male	155 (72.8%)	91 (75.8%)	64 (68.8%)	
Nephrectomy				0.938
Yes	192 (90.1%)	108 (90%)	84 (90.3%)	
No	21 (9.9%)	12 (10%)	9 (9.7%)	
Histology				0.432
Clear cell	164 (77%)	90 (75%)	74 (79.6%)	
Non-clear cell	49 (23%)	30 (25%)	19 (20.4%)	
Tumor grade				0.102
I-II	70 (32.9%)	45 (37.5%)	25 (26.9%)	
III-IV	143 (67.1%)	75 (62.5%)	68 (73.1%)	
eGFR	65 (55–76)	64 (53–75)	67 (55–77)	0.129
Metastatic region sites				
Lung	148 (69.5%)	82 (68.3%)	66 (71%)	0.679
Liver	41 (19.2%)	23 (19.2%)	18 (19.4%)	0.972
Bone	60 (28.2%)	33 (27.5%)	27 (29%)	0.805
Brain	15 (7%)	7 (5.8%)	8 (8.6%)	0.433
IMDC				0.007
Favorable	30 (14.1%)	21 (17.5%)	9 (9.7%)	
Intermediate	117 (54.9%)	72 (60%)	45 (48.4%)	
Poor	66 (31%)	27 (22.5%)	39 (41.9%)	
Treatment response				0.028
Partial response	116 (54.5%)	75 (62.5%)	41 (44.1%)	
Stable disease	64 (30%)	30 (25%)	34 (36.6%)	
Progressive disease	33 (15.5%)	15 (12.5%)	18 (19.4%)	
VB12, pg/mL	306 (179–381)	191 (138–253)	375 (304–517)	
CRP, mg/L	85 (45–125)	50 (30–84)	124 (90–156)	

Abbreviations: BCI: vitamin B12/C-reactive protein index; CRP: C-reactive protein; eGFR: estimated glomerular filtration rate; IMDC: International Metastatic Renal Cell Carcinoma Database Consortium; VB12: vitamin B12.

**Table 2:** Univariate Cox regression analyses of variables associated with PFS and OS.

	PFS		OS	
Variable	HR (95% CI)	*p* Value	HR (95% CI)	*p* Value
Age, years (≥65 vs. <65)	0.87 (0.62–1.21)	0.421	0.99 (0.69–1.41)	0.981
Gender (male vs. female)	1.00 (0.71–1.41)	0.983	1.00 (0.68–1.46)	0.997
Histology (non-clear vs. clear)	1.22 (0.85–1.74)	0.265	1.59 (1.08–2.35)	0.018
eGFR	1.00 (0.98–1.01)	0.683	0.99 (0.95–1.03)	0.819
Nephrectomy (yes vs. no)	0.23 (0.08–0.66)	0.006	2.20 (0.52–9.28)	0.280
Lung metastasis (present vs. absent)	0.75 (0.54–1.04)	0.091	1.01 (0.69–1.47)	0.941
Liver metastasis (present vs. absent)	1.40 (0.96–2.05)	0.077	1.24 (0.81–1.89)	0.311
Bone metastasis (present vs. absent)	1.20 (0.86–1.67)	0.278	1.57 (1.09–2.28)	0.015
Brain metastasis (present vs. absent)	1.12 (0.63–1.99)	0.688	1.98 (1.09–3.60)	0.024
Treatment type		0.442		0.163
Pazopanib	1 (reference)		1 (reference)	
Sunitinib	1.06 (0.77–1.45)	0.704	1.30 (0.91–1.86)	0.146
Cabozantinib	1.51 (0.80–2.86)	0.202	1.73 (0.87–3.40)	0.112
IMDC risk scoring system		<0.001		<0.001
Favorable	1 (reference)		1 (reference)	
Intermediate	1.73 (1.07–2.82)	0.025	2.19 (1.15–4.17)	0.016
Poor	3.92 (2.31–6.67)	<0.001	6.69 (3.41–13.14)	<0.001
BCI (high or low)	1.62 (1.19–2.21)	0.002	2.18 (1.54–3.07)	<0.001

Abbreviations: HR: hazard ratio; CI: confidence interval; IMDC: International Metastatic Renal Cell Carcinoma Database Consortium; BCI: vitamin B12/C-reactive protein index.

**Table 3:** Multivariable Cox regression analyses of variables associated with PFS and OS.

	HR	95% CI		*p* Value
		Lower	Upper	
**PFS**				
Nephrectomy (yes vs. no)	0.12	0.03	0.41	<0.001
Lung metastasis (present vs. absent)	0.61	0.33	1.14	0.127
Liver metastasis (present vs. absent)	1.86	1.00	3.45	0.050
IMDC risk scoring system				<0.001
Favorable risk	1 (reference)			
Intermediate risk	2.22	1.08	4.57	0.030
Poor risk	8.14	3.33	19.89	<0.001
BCI (high or low)	2.40	1.35	4.26	0.003
**OS**				
Histology (non-clear vs. clear)	1.85	1.20	2.85	0.005
Bone metastasis (present vs. absent)	1.55	1.05	2.27	0.025
Brain metastasis (present vs. absent)	2.02	1.09	3.75	0.025
Treatment type				0.204
Pazopanib	1 (reference)			
Sunitinib	1.39	0.96	2.01	0.075
Cabozantinib	1.24	0.62	2.51	0.534
IMDC risk scoring system				<0.001
Favorable	1 (reference)			
Intermediate	2.34	1.21	4.52	0.011
Poor	6.60	3.23	13.11	<0.001
BCI (high or low)	2.01	1.40	2.88	<0.001

Abbreviations: HR: hazard ratio; CI: confidence interval; IMDC: International Metastatic Renal Cell Carcinoma Database Consortium; BCI: vitamin B12/C-reactive protein index.

**Table 4:** Multivariable Cox regression analysis for OS using log-transformed BCI as a continuous variable.

	HR	95% CI		*p* Value
		Lower	Upper	
Treatment type				0.239
Pazopanib	1 (reference)			
Sunitinib	1.36	0.94	1.95	0.097
Cabozantinib	1.33	0.65	2.68	0.426
Histology (non-clear vs. clear)	1.68	1.11	2.55	0.014
Bone metastasis (present vs. absent)	1.62	1.10	2.38	0.013
Brain metastasis (present vs. absent)	1.85	1.00	3.43	0.050
IMDC risk scoring system				<0.001
Favorable	1 (reference)			
Intermediate	2.49	1.29	4.81	0.006
Poor	6.79	3.40	13.50	<0.001
Log-transformed BCI	1.37	1.14	1.65	<0.001

Abbreviations: HR: hazard ratio; CI: confidence interval; IMDC: International Metastatic Renal Cell Carcinoma Database Consortium; BCI: vitamin B12/C-reactive protein index.

## Data Availability

Data are available from the corresponding author on request.

## Supplementary Materials

The following supporting information can be downloaded at: https://www.mdpi.com/article/10.3390/biomedicines14051131/s1, Table S1. Baseline characteristics according to BCI status in the IMDC favorable/intermediate-risk subgroup; Table S2. Baseline characteristics according to BCI status in the IMDC poor-risk subgroup.

## Abbreviations

AUC	Area under the curve
BCI	Vitamin B12/C-reactive protein index
CD	Cluster of differentiation
CI	Confidence interval
CRP	C-reactive protein
DNA	Deoxyribonucleic acid
eGFR	estimated glomerular filtration rate
HR	Hazard ratio
ICI	Immune checkpoint inhibitor
IL	Interleukin
IMDC	International Metastatic Renal Cell Carcinoma Database Consortium
mRCC	Metastatic renal cell carcinoma
OS	Overall survival
PFS	Progression-free survival
RCC	Renal cell carcinoma
ROC	Receiver operating characteristic
TCN1	Transcobalamin I
TNF-α	Tumor necrosis factor-alpha
VB12	Vitamin B12

## References

[1] Siegel R.L., Giaquinto A.N., Jemal A. (2024). Cancer Statistics, 2024. CA Cancer J. Clin..

[2] Padala S.A., Barsouk A., Thandra K.C., Saginala K., Mohammed A., Vakiti A., Rawla P., Barsouk A. (2020). Epidemiology of Renal Cell Carcinoma. World J. Oncol..

[3] Arendt J.F.H., Farkas D.K., Pedersen L., Nexo E., Sørensen H.T. (2016). Elevated Plasma Vitamin B12 Levels and Cancer Prognosis: A Population-Based Cohort Study. Cancer Epidemiol..

[4] Mikkelsen M.K., Lindblom N.A.F., Dyhl-Polk A., Juhl C.B., Johansen J.S., Nielsen D. (2022). Systematic Review and Meta-Analysis of C-Reactive Protein as a Biomarker in Breast Cancer. Crit. Rev. Clin. Lab. Sci..

[5] Socha M.W., Malinowski B., Puk O., Wartęga M., Bernard P., Nowaczyk M., Wolski B., Wiciński M. (2021). C-Reactive Protein as a Diagnostic and Prognostic Factor of Endometrial Cancer. Crit. Rev. Oncol. Hematol..

[6] Kankkunen E., Penttilä P., Peltola K., Bono P. (2022). C-Reactive Protein and Immune-Related Adverse Events as Prognostic Biomarkers in Immune Checkpoint Inhibitor Treated Metastatic Renal Cell Carcinoma Patients. Acta Oncol..

[7] Oh H.K., Lee J.Y., Eo W.K., Yoon S.W., Han S.N. (2018). Elevated Serum Vitamin B _12_ Levels as a Prognostic Factor for Survival Time in Metastatic Cancer Patients: A Retrospective Study. Nutr. Cancer.

[8] Guéant J.-L., Caillerez-Fofou M., Battaglia-Hsu S., Alberto J.-M., Freund J.-N., Dulluc I., Adjalla C., Maury F., Merle C., Nicolas J.-P. (2013). Molecular and Cellular Effects of Vitamin B12 in Brain, Myocardium and Liver through Its Role as Co-Factor of Methionine Synthase. Biochimie.

[9] Volanakis J. (2001). Human C-Reactive Protein: Expression, Structure, and Function. Mol. Immunol..

[10] Frost Z., Bakhit S., Amaefuna C.N., Powers R.V., Ramana K.V. (2025). Recent Advances on the Role of B Vitamins in Cancer Prevention and Progression. Int. J. Mol. Sci..

[11] Kim E.-S., Kim S.Y., Moon A. (2023). C-Reactive Protein Signaling Pathways in Tumor Progression. Biomol. Ther..

[12] Kelly L., White S., Stone P.C. (2007). The B12/CRP Index as a Simple Prognostic Indicator in Patients with Advanced Cancer: A Confirmatory Study. Ann. Oncol..

[13] Montegut C., Correard F., Nouguerède E., Rey D., Chevalier T., Meurer M., Deville J.-L., Baciuchka M., Pradel V., Greillier L. (2021). Prognostic Value of the B12/CRP Index in Older Systemically Treatable Cancer Patients. Cancers.

[14] Tavares F. (2010). Is the B12/CRP Index More Accurate Than You at Predicting Life Expectancy in Advanced Cancer Patients?. J. Pain Symptom Manag..

[15] Heng D.Y.C., Xie W., Regan M.M., Warren M.A., Golshayan A.R., Sahi C., Eigl B.J., Ruether J.D., Cheng T., North S. (2009). Prognostic Factors for Overall Survival in Patients With Metastatic Renal Cell Carcinoma Treated With Vascular Endothelial Growth Factor–Targeted Agents: Results From a Large, Multicenter Study. J. Clin. Oncol..

[16] Couderc A.-L., Puchades E., Villani P., Arcani R., Farnault L., Daumas A., Courcier A., Greillier L., Barlesi F., Duffaud F. (2020). High Serum Vitamin B12 Levels Associated with C-Reactive Protein in Older Patients with Cancer. Oncologist.

[17] Geissbühler P., Mermillod B., Rapin C.-H. (2000). Elevated Serum Vitamin B12 Levels Associated With CRP as a Predictive Factor of Mortality in Palliative Care Cancer Patients. J. Pain Symptom Manag..

[18] Mandaviya P.R., Joehanes R., Brody J., Castillo-Fernandez J.E., Dekkers K.F., Do A.N., Graff M., Hänninen I.K., Tanaka T., AL de Jonge E. (2019). Association of Dietary Folate and Vitamin B-12 Intake with Genome-Wide DNA Methylation in Blood: A Large-Scale Epigenome-Wide Association Analysis in 5841 Individuals. Am. J. Clin. Nutr..

[19] Carmel R., Eisenberg L. (1977). Serum Vitamin B12 and Transcobalamin Abnormalities in Patients with Cancer. Cancer.

[20] Andrès E., Serraj K., Zhu J., Vermorken A.J.M. (2013). The Pathophysiology of Elevated Vitamin B12 in Clinical Practice. QJM Int. J. Med..

[21] Ermens A.A.M., Vlasveld L.T., Lindemans J. (2003). Significance of Elevated Cobalamin (Vitamin B12) Levels in Blood. Clin. Biochem..

[22] Tu Z., He X., Zeng L., Meng D., Zhuang R., Zhao J., Dai W. (2021). Exploration of Prognostic Biomarkers for Lung Adenocarcinoma Through Bioinformatics Analysis. Front. Genet..

[23] Lee Y.-Y., Wei Y.-C., Tian Y.-F., Sun D.-P., Sheu M.-J., Yang C.-C., Lin L.-C., Lin C.-Y., Hsing C.-H., Li W.-S. (2017). Overexpression of Transcobalamin 1 Is an Independent Negative Prognosticator in Rectal Cancers Receiving Concurrent Chemoradiotherapy. J. Cancer.

[24] Liu G., Wang Y., Yue M., Zhao L., Guo Y.-D., Liu Y., Yang H., Liu F., Zhang X., Zhi L. (2020). High Expression of TCN1 Is a Negative Prognostic Biomarker and Can Predict Neoadjuvant Chemosensitivity of Colon Cancer. Sci. Rep..

[25] Zhu X., Yi K., Hou D., Huang H., Jiang X., Shi X., Xing C. (2020). Clinicopathological Analysis and Prognostic Assessment of Transcobalamin I (TCN1) in Patients with Colorectal Tumors. Med. Sci. Monit..

[26] Erkurt M.A., Aydogdu I., Dikilitaş M., Kuku I., Kaya E., Bayraktar N., Ozhan O., Ozkan I., Sönmez A. (2008). Effects of Cyanocobalamin on Immunity in Patients with Pernicious Anemia. Med. Princ. Pract..

[27] Tamura J., Kubota K., Murakami H., Sawamura M., Matsushima T., Tamura T., Saitoh T., Kurabayshi H., Naruse T. (2001). Immunomodulation by Vitamin B12: Augmentation of CD8+ T Lymphocytes and Natural Killer (NK) Cell Activity in Vitamin B12-Deficient Patients by Methyl-B12 Treatment. Clin. Exp. Immunol..

[28] Allin K.H., Nordestgaard B.G. (2011). Elevated C-Reactive Protein in the Diagnosis, Prognosis, and Cause of Cancer. Crit. Rev. Clin. Lab. Sci..

[29] Wu Y., Fu X., Zhu X., He X., Zou C., Han Y., Xu M., Huang C., Lu X., Zhao Y. (2011). Prognostic Role of Systemic Inflammatory Response in Renal Cell Carcinoma: A Systematic Review and Meta-Analysis. J. Cancer Res. Clin. Oncol..

[30] Hu Q., Gou Y., Sun C., Ding W., Xu K., Gu B., Xia G., Ding Q. (2014). The Prognostic Value of C-Reactive Protein in Renal Cell Carcinoma: A Systematic Review and Meta-Analysis. Urol. Oncol. Semin. Orig. Investig..

[31] Yano Y., Ohno T., Komura K., Fukuokaya W., Uchimoto T., Adachi T., Hirasawa Y., Hashimoto T., Yoshizawa A., Yamazaki S. (2022). Serum C-Reactive Protein Level Predicts Overall Survival for Clear Cell and Non-Clear Cell Renal Cell Carcinoma Treated with Ipilimumab plus Nivolumab. Cancers.

[32] Ueda K., Ogasawara N., Yonekura S., Matsunaga Y., Hoshino R., Kurose H., Chikui K., Uemura K., Nakiri M., Nishihara K. (2020). The Prognostic Value of Systemic Inflammatory Markers in Advanced Renal Cell Carcinoma Patients Treated With Molecular Targeted Therapies. Anticancer Res..

[33] Takamatsu K., Mizuno R., Omura M., Morita S., Matsumoto K., Shinoda K., Kosaka T., Takeda T., Shinojima T., Kikuchi E. (2018). Prognostic Value of Baseline Serum C-Reactive Protein Level in Intermediate-Risk Group Patients With Metastatic Renal-Cell Carcinoma Treated by First-Line Vascular Endothelial Growth Factor–Targeted Therapy. Clin. Genitourin. Cancer.

[34] Aktepe O.H., Güner G., Güven D.C., Şahin T.K., Ardiç F.S., Yüce D., Yalçin Ş., Erman M. (2021). The Platelet to Lymphocyte Ratio Predicts Overall Survival Better than the Neutrophil to Lymphocyte Ratio in Metastatic Renal Cell Carcinoma. Turk. J. Med. Sci..

[35] Xu J., Chen P., Cao S., Hu X., Li X. (2024). Prognostic Value of Systemic Immune-Inflammation Index in Patients with Metastatic Renal Cell Carcinoma Treated with Systemic Therapy: A Meta-Analysis. Front. Oncol..

[36] Aktepe O.H., Guven D.C., Sahin T.K., Yildirim H.C., Celikten B., Yeter H.H., Yuce D., Dizdar O., Erman M. (2021). The Predictive Value of Red Blood Cell Distribution Width for Survival Outcomes of Metastatic Renal Cell Carcinoma Patients Treated with Targeted Therapy. Nutr. Cancer.

[37] Kruk L., Mamtimin M., Braun A., Anders H.-J., Andrassy J., Gudermann T., Mammadova-Bach E. (2023). Inflammatory Networks in Renal Cell Carcinoma. Cancers.

[38] Kasherman L., Siu D.H.W., Woodford R., Harris C.A. (2022). Angiogenesis Inhibitors and Immunomodulation in Renal Cell Cancers: The Past, Present, and Future. Cancers.

[39] Khan A.I., Psutka S.P., Patil D.H., Hong G., Williams M.A., Bilen M.A., Sekhar A., Kissick H.T., Narayan V.M., Joshi S.S. (2022). Sarcopenia and Systemic Inflammation Are Associated with Decreased Survival after Cytoreductive Nephrectomy for Metastatic Renal Cell Carcinoma. Cancer.

[40] Lee C.H., Ku J.Y., Seo W.I., Park Y.J., Chung J.I., Kim W., Park T.Y., Ha H.K. (2021). Prognostic Significance of Sarcopenia and Decreased Relative Dose Intensity during the Initial Two Cycles of First-Line Sunitinib for Metastatic Renal Cell Carcinoma. J. Chemother..

[41] Obeid R. (2022). High Plasma Vitamin B12 and Cancer in Human Studies: A Scoping Review to Judge Causality and Alternative Explanations. Nutrients.

[42] Lacombe V., Chabrun F., Lacout C., Ghali A., Capitain O., Patsouris A., Lavigne C., Urbanski G. (2021). Persistent Elevation of Plasma Vitamin B12 Is Strongly Associated with Solid Cancer. Sci. Rep..

[43] Lu Y., Song Y., Xu Y., Ou N., Liang Z., Hu R., Zhang W., Kang J., Wang X., Liu L. (2020). The Prevalence and Prognostic and Clinicopathological Value of PD-L1 and PD-L2 in Renal Cell Carcinoma Patients: A Systematic Review and Meta-Analysis Involving 3,389 Patients. Transl. Androl. Urol..

[44] Peng Y., Dong S., Song Y., Hou D., Wang L., Li B., Wang H. (2021). Key Sunitinib-related Biomarkers for Renal Cell Carcinoma. Cancer Med..

