# Supplementary material for: Elevated B12/CRP Index as a Simple Prognostic Indicator in Patients with Metastatic Renal Cell Carcinoma Treated with First-Line Targeted Therapy
Source: Biomedicines. 2026 May 16;14(5):1131. doi: 10.3390/biomedicines14051131 (PMC13204295; doi:10.3390/biomedicines14051131)
Supplement: Supplementary file 1 [file biomedicines-14-01131-s001.zip › biomedicines-4209736-supplementary.pdf]

Supplementary Table S1. Baseline characteristics according to BCI status in the IMDC favorable/intermediate-risk subgroup.

Characteristics	All patients (n = 147)	Low BCI (n = 93)	High BCI (n = 54)	P value
Age (years)				0.939
<65	104 (70.7%)	66 (71.0%)	38 (70.4%)	
≥65	43 (29.3%)	27 (29.0%)	16 (29.6%)	
Gender				0.872
Female	37 (25.2%)	23 (24.7%)	14 (25.9%)	
Male	110 (74.8%)	70 (75.3%)	40 (74.1%)	
Nephrectomy				0.824
Yes	137 (93.2%)	87 (93.5%)	50 (92.6%)	
No	10 (6.8%)	6 (6.5%)	4 (7.4%)	
Histology				0.319
Clear cell	119 (81%)	73 (78.5%)	46 (85.2%)	
Non-clear cell	28 (19%)	20 (21.5%)	8 (14.8%)	
Tumor grade				0.436
I-II	55 (37.4%)	37 (39.8%)	18 (33.3%)	
III-IV	92 (62.6%)	56 (60.2%)	36 (66.7%)	
eGFR	66 (55-76)	65 (53-74)	69 (55-77)	0.101
Metastatic region sites				
Lung	103 (70.1%)	65 (69.9%)	38 (70.4%)	0.951
Liver	30 (20.4%)	18 (19.4%)	12 (22.2%)	0.678
Bone	38 (25.9%)	26 (28%)	12 (22.2%)	0.444
Brain	9 (6.1%)	5 (5.4%)	4 (7.4%)	0.620

Abbreviations: BCI: vitamin B12/C-reactive protein index; eGFR: estimated glomerular filtration rate; IMDC: International Metastatic Renal Cell Carcinoma Database Consortium.

Supplementary Table S2. Baseline characteristics according to BCI status in the IMDC poor-risk subgroup.

Characteristics	All patients (n = 66)	Low BCI (n = 27)	High BCI (n = 39)	P value
Age (years)				0.522
<65	36 (54.5%)	16 (59.3%)	20 (51.3%)	
≥65	30 (45.5%)	11 (40.7%)	19 (48.7%)	
Gender				0.164
Female	21 (31.8%)	6 (22.2%)	15 (38.5%)	
Male	45 (68.2%)	21 (77.8%)	24 (61.5%)	
Nephrectomy				0.314
Yes	55 (83.3%)	21 (77.8%)	34 (87.2%)	
No	11 (16.7%)	6 (22.2%)	5 (12.8%)	
Histology				0.449
Clear cell	45 (68.2%)	17 (63%)	28 (71.8%)	
Non-clear cell	21 (31.8%)	10 (37%)	11 (28.2%)	
Tumor grade				0.266
I-II	15 (22.7%)	8 (29.6%)	7 (17.9%)	
III-IV	51 (77.3%)	19 (70.4%)	32 (82.1%)	
eGFR	64 (56-72)	61 (56-78)	64 (56-77)	0.548
Metastatic region sites				
Lung	45 (68.2%)	17 (63%)	28 (71.8%)	0.449
Liver	11 (16.7%)	5 (18.5%)	6 (15.4%)	0.737
Bone	22 (33.3%)	7 (25.9%)	15 (38.5%)	0.288
Brain	6 (9.1%)	2 (7.4%)	4 (10.3%)	0.692

Abbreviations: BCI: vitamin B12/C-reactive protein index; eGFR: estimated glomerular filtration rate; IMDC: International Metastatic Renal Cell Carcinoma Database Consortium.
